# Fast Grid-Based Ground Segmentation of LiDAR Point Clouds Using Dual-Seed Expansion

**DOI:** 10.3390/s26144603

**Published:** 2026-07-20

**Authors:** Jongyun Lee, Manbok Park

**Affiliations:** Department of Electronics Engineering, Korea National University of Transportation, Chungju-si 27469, Republic of Korea; zx113355@naver.com

**Keywords:** LiDAR point cloud, ground segmentation, grid-based segmentation, autonomous driving, SemanticKITTI

## Abstract

Ground segmentation is an essential preprocessing step for LiDAR-based perception in autonomous driving, as it directly affects subsequent object clustering and obstacle detection. This paper proposes a fast grid-based ground segmentation method for 3D Light Detection and Ranging (LiDAR) point clouds using dual-seed expansion. The input point cloud is projected onto a two-dimensional Cartesian grid, where cell-wise height statistics, including minimum height, maximum height, mean height, and height variance, are computed. Initial ground candidates are selected by combining a global height-distribution-based seed and a LiDAR-mounting-height-based seed to reduce missed ground candidates in sloped or sparse regions. Ground cells are then expanded to nearby cells based on the local height difference. To reduce misclassification in cells containing both ground and non-ground points, a local height-band criterion is applied for point-level refinement. The proposed method was implemented in Robot Operating System 1 (ROS 1) and evaluated on the SemanticKITTI dataset. Experimental results showed an average accuracy of 96.34%, an F1-score of 97.11%, and a ground Intersection over Union (IoU) of 94.40%. The average processing time over SemanticKITTI sequences 00–10 was 12.15 ms per frame. These results demonstrate that the proposed method is suitable as a fast preprocessing module for LiDAR-based autonomous driving perception.

## 1. Introduction

Autonomous vehicles use various sensors, such as cameras, radar, and LiDAR, to perceive their surrounding environments. Among these sensors, LiDAR provides three-dimensional distance information in the form of point clouds and is widely used for object detection, obstacle detection, localization, and drivable-area analysis [1]. However, raw LiDAR point clouds contain a large number of ground points originating from roads, sidewalks, parking areas, lane markings, and terrain surfaces. These ground points are often regarded as background in subsequent perception tasks and may increase the computational burden of object clustering and obstacle detection. Therefore, ground segmentation is an important preprocessing step in LiDAR-based autonomous driving perception systems.

LiDAR ground segmentation has been studied using various approaches, including point-cloud-based methods, range-image-based methods, Cartesian-grid-based methods, polar-grid-based methods, and learning-based methods [2]. Point-cloud-based methods directly use the original three-dimensional coordinates of input points to estimate ground surfaces through plane fitting, local surface estimation, or geometric relationships between neighboring points [3,4,5,6,7]. These methods can exploit the geometric structure of point clouds directly, but their computational cost generally increases as the number of points increases. In addition, when the ground surface is approximated by a single plane or a limited number of planes, segmentation accuracy may degrade in real road environments containing slopes, curbs, discontinuous road structures, or multiple height levels.

Triangulated Irregular Network (TIN)-based ground filtering methods construct a triangulated irregular network from selected low points and progressively refine the ground surface model [8]. These methods can represent terrain surfaces with irregular structures, but they generally require iterative surface construction and refinement, which may increase computational cost for real-time LiDAR preprocessing. In contrast, the proposed method does not explicitly construct a terrain surface model and instead performs frame-wise ground segmentation using simple cell-wise height statistics and local height-continuity-based expansion.

To reduce the computational burden of point-wise processing, several methods transform LiDAR point clouds into structured two-dimensional representations. Range-image-based methods convert point clouds into cylindrical range images and perform segmentation using the neighborhood relationship between adjacent scan lines or image pixels [9,10,11,12]. These methods can achieve high processing speed because they exploit the inherent scanning structure of LiDAR sensors. However, their performance and implementation are affected by sensor-specific characteristics, such as the number of LiDAR channels, vertical angle distribution, and horizontal angular resolution. In addition, because range-image-based methods mainly use scan-line or image-domain connectivity, additional processing may be required when grid-based terrain information is needed for autonomous driving modules.

Grid-based methods project LiDAR point clouds onto a two-dimensional plane and classify ground regions using cell-wise height statistics or local geometric features [13,14,15]. These methods reduce point-wise computation to cell-wise computation and provide a structured representation of the surrounding environment. In particular, Cartesian-grid-based methods divide the region of interest into fixed-size rectangular cells in the x-y plane. Each cell stores statistics such as minimum height, maximum height, mean height, height variance, and point count. This structure is simple to implement, efficient in memory access, and suitable for parallel processing. GroundGrid is a representative Cartesian-grid-based method that performs ground segmentation and terrain estimation using a two-dimensional elevation map [13]. It reports high ground segmentation performance and real-time processing capability on the SemanticKITTI dataset [13,16,17].

However, Cartesian-grid-based methods also have limitations. Because a fixed grid resolution is used over the entire region of interest, the distance-dependent sparsity of LiDAR points cannot be fully reflected. In far regions, the number of points in each cell decreases, which can make height statistics unstable. Conversely, if the grid resolution is increased to improve stability in sparse regions, ground and non-ground points can be included in the same cell in near regions. This mixed-cell problem may cause low obstacles, curbs, vegetation boundaries, or object boundary points to be misclassified as ground. In addition, the selection of initial ground candidates strongly affects the final segmentation result. If too few initial ground cells are selected, actual ground regions may remain unclassified. Conversely, if non-ground cells are included as initial ground candidates, the error can propagate during the ground expansion process.

Polar-grid-based methods have been proposed to alleviate the limitations caused by non-uniform LiDAR point density [18,19,20,21]. These methods divide the surrounding area into radial and angular regions centered on the LiDAR sensor or vehicle, which better reflects the actual measurement characteristics of rotating LiDAR sensors. Because the cell size naturally increases with distance, polar-grid-based methods can handle sparse points in far regions more effectively than fixed Cartesian grids. Representative methods, including region-wise ground estimation and concentric-zone-based approaches, estimate local ground models in divided regions and show robust performance in complex urban environments [19,20]. However, polar-grid-based methods often require additional parameters for radial intervals, angular sectors, zone division, and local ground model estimation. Their implementation can also become more complex than that of Cartesian-grid-based methods.

Recently, learning-based methods have also been applied to LiDAR ground estimation and point cloud segmentation [22,23,24,25,26,27,28]. These methods can learn complex ground and object patterns from labeled datasets and have the potential to improve robustness in challenging environments. However, they generally require a large amount of annotated training data, additional computational resources, and model optimization for real-time deployment. Therefore, simple rule-based or statistics-based methods are still useful in autonomous driving systems with limited computational resources.

To address these issues, this paper proposes a fast grid-based LiDAR point cloud ground segmentation method using dual-seed expansion. The proposed method maintains the simplicity and computational efficiency of Cartesian-grid-based representation while improving initial ground candidate selection and mixed-cell handling. The input point cloud is first projected onto a two-dimensional Cartesian grid, and cell-wise height statistics are computed. Initial ground candidates are then selected using two complementary criteria: a global height-distribution-based seed and a LiDAR-mounting-height-based seed. The global seed captures relatively low regions in the scene, while the sensor-height seed reflects the expected ground height in the vehicle coordinate system. By combining these two criteria, the proposed method reduces missed ground candidates in sloped or sparse regions.

After dual-seed initialization, ground regions are expanded to neighboring cells based on height continuity. This expansion process uses the spatial continuity of ground surfaces while maintaining a simple grid-based structure. Finally, to reduce misclassification in cells containing both ground and non-ground points, a local height-band criterion is applied at the point level. The proposed method is implemented in ROS1 and evaluated on the SemanticKITTI dataset to analyze both ground segmentation accuracy and processing time.

The main contribution of this study is a fast frame-wise Cartesian-grid-based ground segmentation method that improves initial ground candidate selection using dual-seed initialization and reduces mixed-cell errors using point-level height-band refinement. Unlike elevation-map-based approaches, the proposed method does not rely on temporal map updates, terrain interpolation, or learning-based inference, which makes the algorithm simple and computationally efficient for LiDAR preprocessing.

The remainder of this paper is organized as follows. Section 2 describes the proposed method, including grid generation, cell-wise height statistics, dual-seed initialization, neighbor-cell expansion, and mixed-cell refinement. Section 3 presents the experimental setup and evaluation results using the SemanticKITTI dataset. Section 4 discusses the accuracy–speed trade-off, limitations, and applicability of the proposed method. Section 5 concludes the paper and outlines future research directions.

## 2. Materials and Methods

### 2.1. Overview of the Proposed Method

The proposed method performs fast ground segmentation of 3D LiDAR point clouds using a Cartesian grid representation and dual-seed expansion. The overall procedure consists of five main steps: grid generation, cell-wise height statistics computation, dual-seed initialization, neighbor-cell expansion, and point-level refinement for mixed cells.

**Figure 1:**
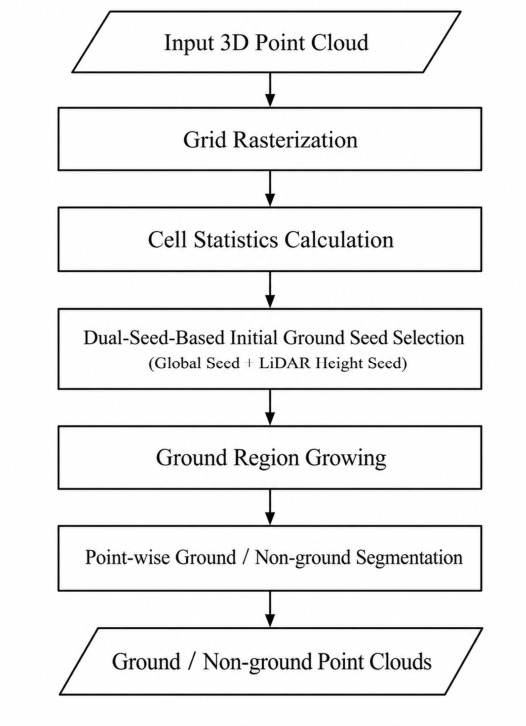
Overall structure of the proposed algorithm.

First, the input LiDAR point cloud is rasterized onto a two-dimensional Cartesian grid in the vehicle coordinate system. For each grid cell, height statistics, including minimum height, maximum height, mean height, height variance, and the number of points, are computed. Initial ground candidate cells are selected using two complementary criteria: a global height-distribution-based seed and a LiDAR-mounting-height-based seed. The selected seed cells are used as initial ground cells, and neighboring cells are iteratively expanded according to height continuity. Finally, for cells containing both ground and non-ground points, a local height-band criterion is applied to refine the classification at the point level. The final output consists of ground and non-ground point clouds. Figure 1 shows the overall structure of the proposed algorithm, including grid generation, cell-wise height statistics computation, dual-seed initialization, neighbor-cell expansion, and point-level refinement.

### 2.2. Grid Representation of LiDAR Point Clouds

Let the input LiDAR point cloud be defined as

(1) $$P=\left\{p_{i}\;\right|\;p_{i}=\left(x_{i},\;y_{i},\;z_{i}\right)\;i=1,\;2,\;\ldots ,\;N,\}$$

where P denotes a single-frame LiDAR point cloud, $p_{i}$ is the i-th point, and $x_{i}$, $y_{i}$ and $z_{i}$ represent the three-dimensional coordinates of the point in the vehicle coordinate system. N is the total number of points in the input scan. The region of interest (ROI) is defined by the ranges [$x_{min},\;x_{max}$] and [$y_{min},\;y_{max}$]. Points outside this ROI are excluded from the grid construction process. The remaining points are projected onto a two-dimensional Cartesian grid with a grid resolution r. The grid indices of a point $p_{i}$ are computed as

(2) $$g_{x}\left(i\right)=\left\lfloor \frac{x_{i}-x_{min}}{r}\right\rfloor$$

(3) $$g_{y}\left(i\right)=\left\lfloor \frac{y_{i}-y_{min}}{r}\right\rfloor$$

where $g_{x}\left(i\right)$ and $g_{y}\left(i\right)$ denote the grid indices in the x- and y-directions, respectively. The floor operator (⌊⋅⌋) is used to convert the continuous point coordinates into discrete grid indices.

The total number of grid cells in the x- and y-directions is given by

(4) $$W=\left\lceil \frac{x_{max}-x_{min}}{r}\right\rceil$$

(5) $$H=\left\lceil \frac{y_{max}-y_{min}}{r}\right\rceil$$

where W and H denote the grid width and height, respectively, and (⌈⋅⌉) denotes the ceiling operator. Each valid point is assigned to the corresponding grid cell $C_{\left\{u,v\right\}}$, where u = $g_{x}\left(i\right)$ and v = $g_{y}\left(i\right)$.

**Figure 2:**
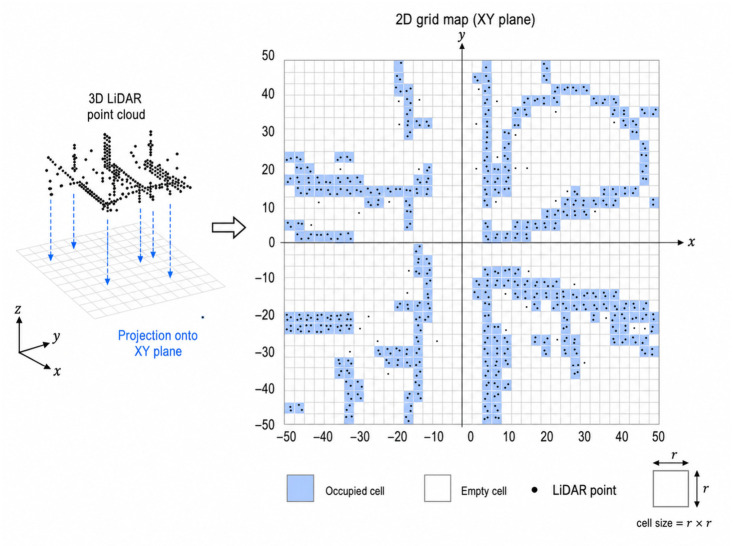
Projection of a 3D LiDAR Point Cloud onto a Two-Dimensional Cartesian Grid. The arrow indicates the transformation from the 3D LiDAR point cloud to the 2D Cartesian grid, and the blue dashed lines indicate the projection of individual points onto the XY plane.

The Cartesian grid representation reduces point-wise operations to cell-wise operations and provides a structured form that is efficient for memory access and parallel processing. Figure 2 illustrates the projection of a 3D LiDAR point cloud onto a two-dimensional Cartesian grid. Each point is assigned to a grid cell according to its x- and y-coordinates, and cells containing one or more points are treated as occupied cells.

### 2.3. Cell-Wise Height Statistics

For each grid cell $C_{\left\{u,v\right\}}$, the set of points contained in the cell is defined as

(6) $$P_{\left\{u,v\right\}}=\left\{p_{i}\;\in \;P\;\right|g_{x}\left(i\right)=u,\;g_{y}\left(i\right)=v\}$$

Let $n_{\left\{u,v\right\}}$ be the number of points in $P_{\left\{u,v\right\}}$. For non-empty cells, the following height statistics are computed using the z-coordinates of the points:

(7) $$z_{min}\;\left(u,v\right)\;=\min z_{i}\;\left\{p_{i}\;\;\in \;P_{\left\{u,v\right\}}\;\right\}\;$$

(8) $$z_{max}\left(u,v\right)=\max z_{i}\;\left\{p_{i}\;\;\in \;P_{\left\{u,v\right\}}\;\right\}$$

(9) $$z_{mean}\left(u,v\right)=\frac{1}{n_{\left\{u,v\right\}}}\;\Sigma \;z_{i}\;\left\{p_{i}\in \;P_{\left\{u,v\right\}}\right\}$$

(10) $$\sigma_{z}^{2}\;(u,v)=\frac{1}{n_{\left\{u,v\right\}}}\;\Sigma_{\left\{p_{i}\in \;P_{\left\{u,v\right\}}\right\}}\;{\left(z_{i}-z_{mean}\left(u,v\right)\right)}^{2}\;$$

**Figure 3:**
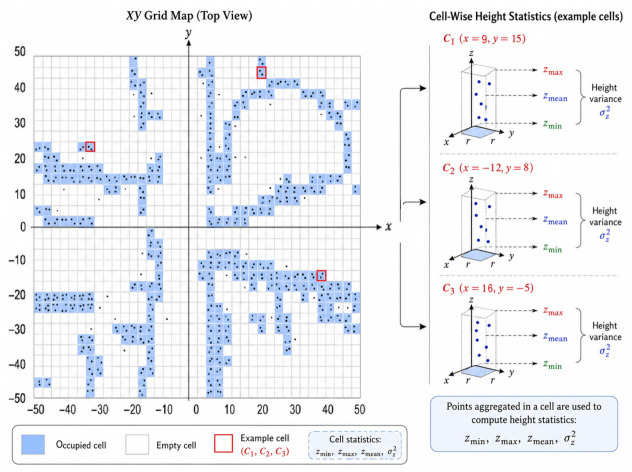
Cell-Wise Height Statistics in the Cartesian Grid. The black dots represent LiDAR points.

In general, ground cells tend to have a small height range and low height variance, whereas cells containing vehicles, curbs, vegetation, or other non-ground objects may have a larger height range or higher variance. Therefore, the proposed method uses the minimum height and height variance of each cell to determine initial ground candidates and to perform subsequent ground expansion. Figure 3 shows the cell-wise height statistics computation process. For each occupied cell, the minimum height, maximum height, mean height, and height variance are computed from the z-coordinates of the points inside the cell.

### 2.4. Dual-Seed Initialization

The selection of initial ground candidates is critical in grid-based ground segmentation. If too few ground cells are selected at the initial stage, actual ground regions may not be sufficiently expanded. Conversely, if non-ground cells are selected as initial ground candidates, the error can propagate to neighboring cells during expansion.

To improve the robustness of initial ground candidate selection, this study uses a dual-seed initialization strategy. The proposed strategy combines a global height-distribution-based seed and a LiDAR-mounting-height-based seed.

#### 2.4.1. Global Height-Distribution-Based Seed

The global seed is determined using the distribution of minimum heights in valid grid cells. First, the minimum height values of all non-empty cells are collected. A global reference height $z_{g}$ is then determined as the q-th percentile of the collected minimum height values:

(11) $$z_{g}\;=\;percentile\left(\left\{z_{min}\;\left(u,v\right)\right\}\;,\;q\right)$$

where q denotes the lower percentile ratio. The global seed is designed to select relatively low regions in the current scene as ground candidates.

**Figure 4:**
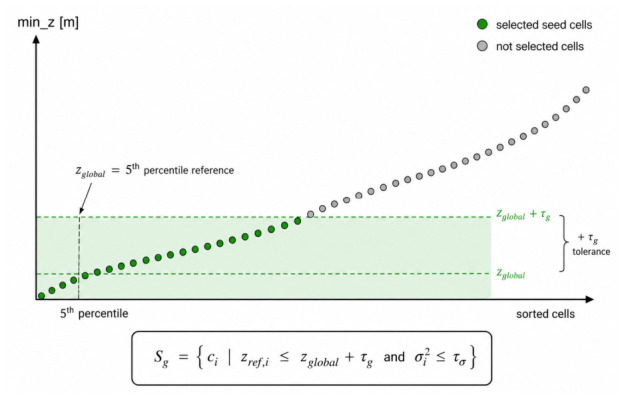
Global Height-Distribution-Based Seed Selection.

A grid cell $C_{\left\{u,v\right\}}$ is selected as a global seed if it satisfies the following conditions:

(12) $$z_{min}\left(u,v\right)\;\;\leq \;z_{g}+T_{g}$$

(13) $$\sigma_{z}^{2}\left(u,v\right)\;\leq \;T_{var}$$

where $T_{g}$ is the height tolerance threshold for the global seed, and $T_{var}$ is the height variance threshold. Figure 4 illustrates the global height-distribution-based seed selection process. The lower percentile of the cell minimum-height distribution is used to determine the global reference height, and cells satisfying both the height and variance conditions are selected as global seed cells.

#### 2.4.2. LiDAR-Mounting-Height-Based Seed

The global seed can reflect the relative height distribution of the entire scene. However, in sloped or sparse regions, a single global height criterion may not provide sufficient initial ground candidates. To compensate for this limitation, the proposed method additionally uses the LiDAR mounting height.

If the LiDAR sensor is mounted at a height $h_{L}$ above the ground, the expected ground height in the vehicle coordinate system can be approximated as

(14) $$z_{s}=-h_{L}$$

**Figure 5:**
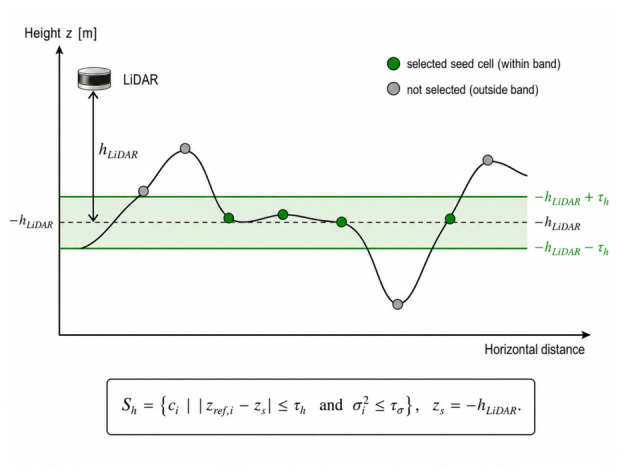
LiDAR-Mounting-Height-Based Seed Selection.

A grid cell $C_{\left\{u,v\right\}}$ is selected as a sensor-height seed if its minimum height is close to the expected ground height:

(15) $${|z}_{min}\left(u,v\right)\;-\;z_{s}|\;\leq T_{s}$$

(16) $$\sigma_{z}^{2}\left(u,v\right)\;\leq \;T_{var}$$

where $T_{s}$ is the height tolerance threshold for the sensor-height seed. Figure 5 illustrates the LiDAR-mounting-height-based seed selection process. The expected ground height is approximated from the LiDAR mounting height in the vehicle coordinate system, and cells whose minimum height is close to this expected ground height are selected as sensor-height seed cells.

The LiDAR-mounting-height-based seed was introduced to compensate for the limitation of using only the global height distribution. In sloped roads or sparse distant regions, the lower percentile of the cell minimum-height distribution may be biased toward only a limited part of the scene, and some actual ground cells may not satisfy the global seed condition. The LiDAR-height-based seed compensates for this limitation by selecting additional cells whose minimum height is close to the expected ground height in the vehicle coordinate system.

#### 2.4.3. Combination of Dual Seeds

The final initial ground candidate set is defined as the union of the global seed set and the sensor-height seed set:

(17) $$S_{0}\;=\;S_{g}\;\cup \;S_{s}$$

where $S_{g}$ denotes the set of cells satisfying the global seed condition, and $S_{s}$ denotes the set of cells satisfying the sensor-height seed condition.

By combining these two seed criteria, the proposed method uses both scene-level height distribution and sensor-level prior information. This reduces missed ground candidates in sloped or sparse regions while maintaining a simple cell-wise decision structure. The two seed sets illustrated in Figure 4 and Figure 5 are combined before the neighbor-cell expansion step.

### 2.5. Neighbor-Cell Expansion Based on Height Continuity

After the initial ground candidate cells are selected, the ground region is expanded using height continuity between neighboring cells. Ground surfaces generally form spatially continuous regions. Therefore, if the height difference between a ground cell and its neighboring cell is sufficiently small, the neighboring cell can also be regarded as ground.

The proposed method uses eight-neighbor connectivity, including horizontal, vertical, and diagonal neighboring cells. All cells in $S_{0}$ are inserted into a queue and used as the initial frontier for expansion. For each ground cell $C_{\left\{u,v\right\}}$ removed from the queue, its neighboring cell $C_{\left\{m,n\right\}}$ is examined. If the neighboring cell is empty or has already been classified as ground, it is skipped.

The height difference between the current ground cell $C_{\left\{u,v\right\}}$ and the neighboring cell $C_{\left\{m,n\right\}}$ is defined as

(18) $$\Delta z=|z_{min}\left(m,n\right)-z_{min}\left(u,v\right)|$$

The planar distance between the two cells is defined as

(19) d=r

for horizontal and vertical neighbors, and

(20) $$d=\sqrt{2}r$$

for diagonal neighbors. The local slope between the two cells is then computed as

(21) s=Δz / d

The neighboring cell $C_{\left\{m,n\right\}}$ is classified as ground if

(22) $$s\;\leq \;T_{slope}$$

where $T_{slope}$ is the slope threshold for neighbor-cell expansion.

**Figure 6:**
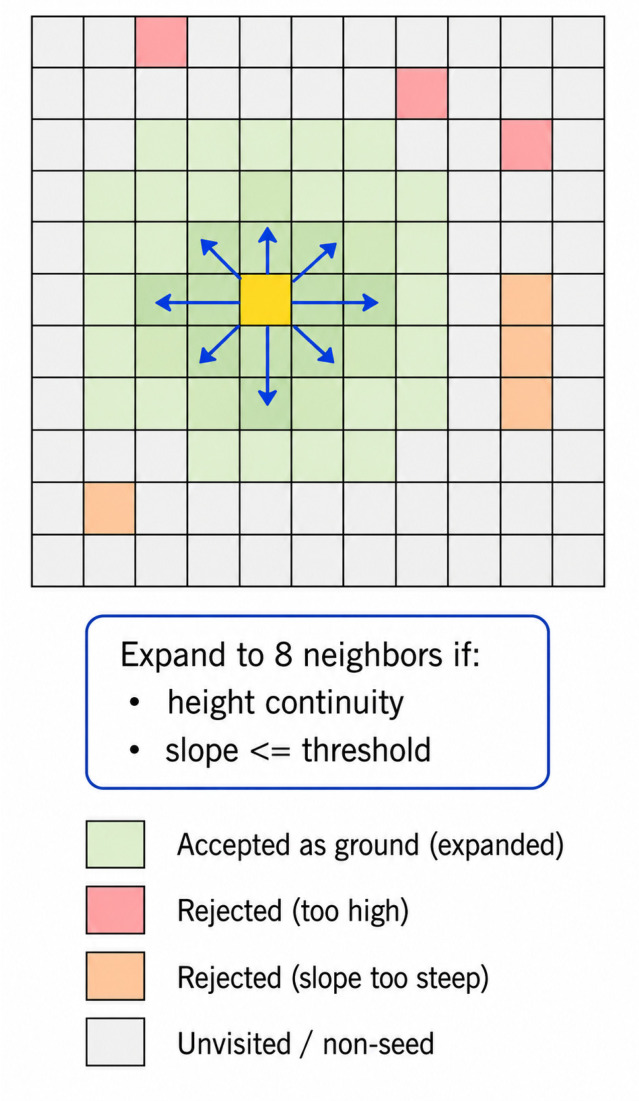
Eight-Neighbor Ground Cell Expansion.

When a neighboring cell is newly classified as ground, it is inserted into the queue and used as a new expansion frontier. This process is repeated until the queue becomes empty. Through this iterative expansion, the proposed method extends ground regions from reliable initial seeds to neighboring cells with smooth height transitions. Figure 6 shows the eight-neighbor ground cell expansion process. Starting from the initial seed cells, neighboring cells are accepted as ground when the height-continuity and slope conditions are satisfied.

In this study, the local slope does not represent the global road inclination. It is used as a local height-continuity criterion between adjacent grid cells. Therefore, a neighboring cell is accepted as ground when the height difference between the current ground cell and the neighboring cell is sufficiently small relative to their planar distance.

### 2.6. Point-Level Refinement for Mixed Cells

After the neighbor-cell expansion is completed, each point is initially classified according to the ground label of its corresponding grid cell. If a point belongs to a cell classified as ground, it is assigned to the ground point cloud. Otherwise, it is assigned to the non-ground point cloud.

However, a single grid cell may contain both ground and non-ground points, especially near curbs, vehicles, vegetation, or object boundaries. If all points in such a cell are classified only by the cell label, non-ground points may be incorrectly included in the ground class. To alleviate this mixed-cell problem, the proposed method applies a point-level refinement process using a local height-band criterion.

For each ground cell $C_{\left\{u,v\right\}}$, the internal height range is computed as

(23) $$R_{z}\left(u,v\right)=z_{max}\left(u,v\right)-z_{min}\left(u,v\right)$$

If the height range is larger than a predefined threshold $T_{mix}$,

(24) $$R_{z}\left(u,v\right)>T_{mix}$$

the cell is regarded as a mixed cell. In this case, a point $p_{i}$ ∈ $P_{\left\{u,v\right\}}$ is classified as ground only if its height is close to the minimum height of the cell:

(25) $$z_{i}\;-\;z_{min}\left(u,v\right)\;\leq \;T_{band}$$

where $T_{band}$ is the local height-band threshold. Points that do not satisfy this condition are classified as non-ground.

**Figure 7:**
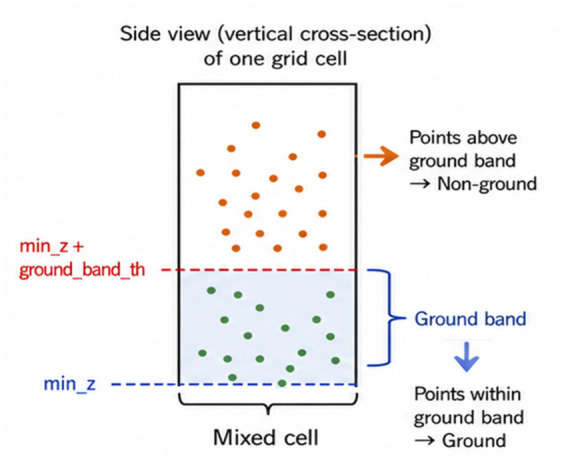
Mixed-Cell Separation Using a Local Height Band.

For cells that are classified as ground but do not satisfy the mixed-cell condition, all points in the cell are classified as ground. This refinement process reduces the misclassification of non-ground points in cells containing both ground and object points. Figure 7 illustrates the point-level refinement process for mixed cells. When ground and non-ground points coexist within the same grid cell, only points within the local ground height band are classified as ground, while points above the band are classified as non-ground.

## 3. Results

The proposed method was implemented in ROS1 using C++ and evaluated on the SemanticKITTI dataset. SemanticKITTI provides point-wise semantic labels for KITTI odometry sequences and is widely used for evaluating LiDAR-based semantic scene understanding. In this study, the semantic labels were converted into binary ground and non-ground classes for ground segmentation evaluation.

The ground class was defined using the road, sidewalk, parking, lane-marking, other-ground, and terrain labels. The remaining labels, such as vehicles, pedestrians, buildings, poles, traffic signs, and other non-ground structures, were treated as non-ground. Vegetation, unlabeled points, and outlier points were excluded from the evaluation following the evaluation setting used in GroundGrid.

The proposed algorithm processes each LiDAR frame independently and does not maintain a temporal elevation map. The input point cloud was segmented into ground and non-ground point clouds for each frame. The processing time was measured from the beginning of the LiDAR callback to the completion of ground and non-ground point cloud generation. All parameters of the proposed method were fixed during the evaluation.

Table 1 and Table 2 summarize the experimental environment and the parameters used in the experiments, respectively.

### 3.1. Evaluation Metrics

The ground segmentation performance was evaluated using accuracy, precision, recall, F1-score, and intersection over union (IoU). True positive (TP) denotes the number of ground points correctly classified as ground, while false positive (FP) denotes the number of non-ground points incorrectly classified as ground. False negative (FN) denotes the number of ground points incorrectly classified as non-ground, and true negative (TN) denotes the number of non-ground points correctly classified as non-ground.

Accuracy measures the ratio of correctly classified points among all evaluated points. Precision measures the ratio of correctly classified ground points among all points predicted as ground. Recall measures the ratio of correctly classified ground points among all actual ground points. The F1-score is the harmonic mean of precision and recall. IoU measures the overlap between the predicted ground points and the ground-truth ground points.

### 3.2. Quantitative Evaluation of Ground Segmentation

Table 3 presents the quantitative ground segmentation results on SemanticKITTI sequences 00 to 10. The results of Patchwork++, Jump Point Convolution (JPC), and GroundGrid are cited from Steinke et al. [13], while the results of the proposed method were obtained in this study. The proposed method achieved an average accuracy of 96.34%, precision of 96.61%, recall of 97.63%, F1-score of 97.11%, and ground IoU of 94.40%.

The results show that the proposed method maintained reliable ground segmentation performance across most sequences. The highest ground IoU was observed on sequence 04, where the proposed method achieved 97.11%. In contrast, sequence 10 showed the lowest ground IoU of 90.04%. This result indicates that complex terrain structures, vegetation boundaries, and abrupt height changes can affect the performance of the proposed Cartesian-grid-based expansion process.

Compared with GroundGrid, the proposed method showed lower average segmentation accuracy. GroundGrid achieved an average accuracy of 96.60%, F1-score of 97.32%, and ground IoU of 94.78%, which were higher than those of the proposed method. This difference can be attributed to the fact that GroundGrid performs terrain estimation using elevation map update, ground confidence estimation, interpolation, and point cloud segmentation. In contrast, the proposed method focuses on fast frame-wise ground segmentation using simple cell-wise statistics, dual-seed initialization, and height-continuity-based expansion.

### 3.3. Ablation Study

An ablation study was conducted to analyze the contribution of each component of the proposed method. The evaluated variants were global seed only, LiDAR-height seed only, dual seed without neighbor expansion, dual seed without mixed-cell refinement, and the complete proposed method. All experiments were conducted on SemanticKITTI sequences 00–10 using the same region of interest, grid resolution, evaluation protocol, and hardware environment.

Table 4 shows the ablation study results. The global seed only variant achieved an F1-score of 91.88% and an IoU(G) of 85.20%. Although its precision remained high, its recall decreased to 87.63%. This result indicates that using only the global height-distribution-based seed does not provide sufficient initial ground candidates in some scenes, especially when the ground height distribution varies due to slopes, sparse regions, or discontinuous terrain.

The LiDAR-height seed only variant achieved an F1-score of 96.79% and an IoU(G) of 93.80%, which were close to the results of the complete proposed method. This result shows that the LiDAR-height seed provides a strong prior for selecting ground candidates in the vehicle coordinate system. However, the complete dual-seed configuration further improved the F1-score and IoU(G), indicating that the global seed can complement the LiDAR-height seed in scenes where the expected sensor-height-based ground level alone is insufficient.

The dual seed without neighbor expansion variant achieved very high precision of 99.78%, but its recall decreased to 78.12%. This means that the selected seed cells were reliable, but the ground regions were not sufficiently extended to neighboring cells. Therefore, neighbor-cell expansion is necessary to recover continuous ground regions from the initial seed cells.

The dual seed without mixed-cell refinement variant showed the highest recall of 98.91%, but its precision decreased significantly to 72.26%. Its IoU(G) also decreased to 71.70%. This result indicates that when all points in ground-labeled cells are directly classified as ground, non-ground points inside mixed cells are frequently misclassified as ground. Therefore, the point-level mixed-cell refinement is essential for reducing false positives near curbs, vehicles, vegetation boundaries, and object boundaries.

The complete proposed method achieved the best overall performance, with an accuracy of 96.40%, an F1-score of 97.16%, and an IoU(G) of 94.49%. These results demonstrate that dual-seed initialization, neighbor-cell expansion, and mixed-cell refinement each contribute to stable ground segmentation. In particular, the ablation results show that the proposed method improves the balance between precision and recall.

### 3.4. Parameter Sensitivity Analysis

The parameter sensitivity analysis was conducted to examine the influence of the main threshold values on the ground segmentation performance. In each experiment, one parameter was varied while the other parameters were fixed. The evaluated parameters included the grid resolution, LiDAR-height seed threshold, local slope threshold, mixed-cell height range threshold, and local ground height-band threshold. The results are summarized in Table 5, Table 6, Table 7, Table 8 and Table 9. The grid-resolution sensitivity analysis was conducted using the same parameter-sensitivity evaluation setting to analyze relative trends. Therefore, the values in Table 5 are used to show the effect of grid resolution, while the final performance of the proposed method is reported in Table 3.

Among the evaluated parameters, the grid resolution has the most direct influence on both segmentation accuracy and processing time. Therefore, its effect was analyzed in more detail using both a sensitivity table and a Pareto-style trade-off plot.

**Figure 8:**
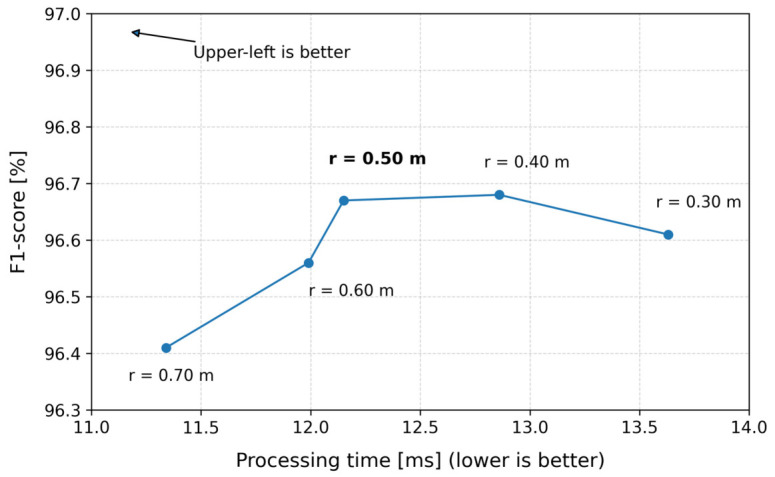
F1-score–runtime trade-off by grid resolution. The bold text indicates the selected grid resolution used in the subsequent experiments.

Figure 8 shows the trade-off between segmentation accuracy and processing time for different grid resolutions of the proposed method. Smaller grid cells slightly improved the segmentation accuracy but increased the runtime, whereas larger grid cells reduced the runtime at the cost of a slight decrease in accuracy. The selected grid resolution of 0.50 m provided a balanced trade-off between the two.

Overall, the proposed method showed stable performance near the selected parameter values. The grid resolution affected both accuracy and processing time, where smaller cells slightly improved segmentation accuracy but increased runtime. The LiDAR-height seed threshold and local slope threshold showed only marginal performance differences around the selected values, indicating that the proposed dual-seed expansion is not highly sensitive to small changes in these thresholds. The mixed-cell height range threshold and local ground height-band threshold affected the balance between preserving ground points and suppressing false positives in mixed cells. Based on these results, the final parameter values were selected to provide stable segmentation performance while maintaining computational efficiency.

### 3.5. Qualitative Evaluation

**Figure 9:**
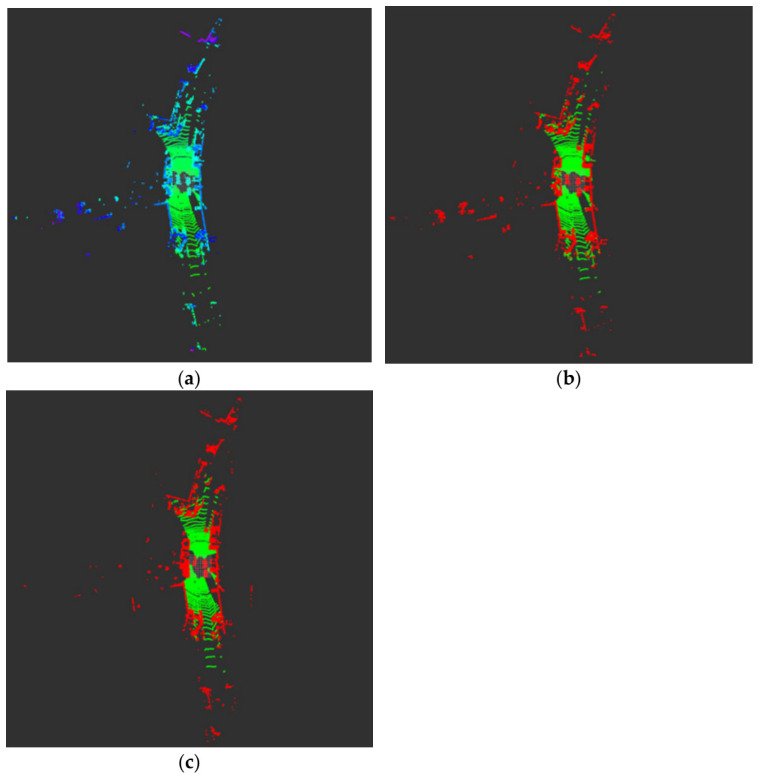
Qualitative ground segmentation result on the SemanticKITTI dataset: (**a**) Input LiDAR point cloud; (**b**) Ground segmentation result of the proposed method; (**c**) Ground-truth ground labels. In (**a**), point colors represent height, where lower points are shown in green and higher points gradually change toward purple. In (**b**,**c**), ground points are shown in green and non-ground points are shown in red.

Figure 9 shows a qualitative example of the proposed ground segmentation result on the SemanticKITTI dataset. Figure 9a presents the input LiDAR point cloud, Figure 9b presents the ground segmentation result of the proposed method, and Figure 9c presents the corresponding ground-truth ground labels. In the visualization, ground points are shown in green and non-ground points are shown in red. Overall, the proposed method separated road surfaces and nearby flat ground regions from non-ground objects in most areas. Vehicles, walls, and other elevated structures were generally classified as non-ground, while road surfaces and adjacent flat regions were classified as ground.

**Figure 10:**
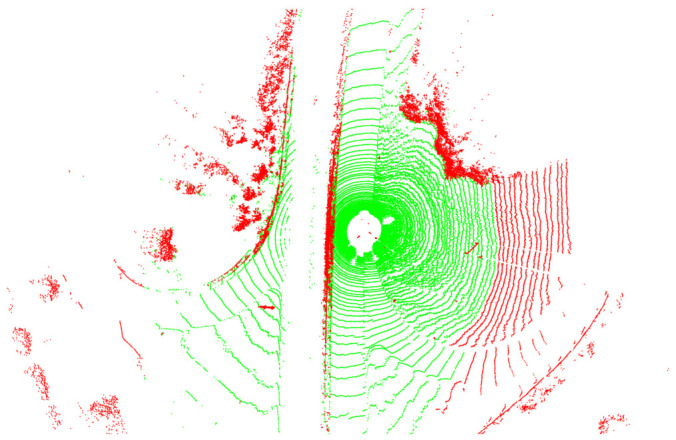
Ground segmentation result of the proposed method in sequence 01. Ground points are shown in green and non-ground points are shown in red.

**Figure 11:**
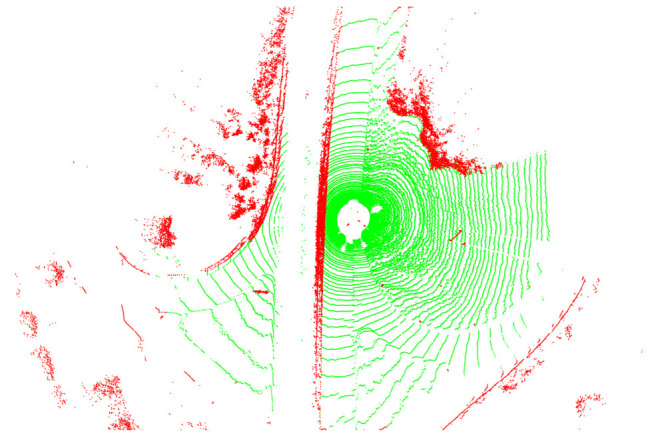
Ground-truth ground labels in sequence 01. Ground points are shown in green and non-ground points are shown in red.

Figure 10 and Figure 11 show a representative challenging case from sequence 01. Compared with the ground-truth labels, the proposed method correctly segmented most of the near-range road surface, but some ground points in the sparse distant region were classified as non-ground. This tendency is especially visible on the right side of the scene, where the point density becomes lower as the distance from the LiDAR sensor increases. In such regions, the local height-continuity assumption between neighboring grid cells becomes less reliable, and the ground expansion process may not fully recover all actual ground cells.

This qualitative observation is consistent with the characteristics of the proposed method. Because the method relies on fixed-resolution Cartesian grid cells and local height-continuity-based expansion, its performance can become less stable in sparse distant regions or around locally discontinuous road-side structures. Nevertheless, the overall qualitative results indicate that the proposed method provides reliable ground segmentation in typical road scenes while maintaining a simple and fast processing structure.

### 3.6. Processing Time Evaluation

The processing time was evaluated on SemanticKITTI sequences 00–10 to analyze the computational efficiency of the proposed method. For the processing-time evaluation, all methods received input point clouds pre-cropped to the same Cartesian ROI of −50 m to 50 m in both the x- and y-directions. The cropping operation was performed before the point cloud was passed to each method and was not included in the measured runtime. The processing time represents the time required to receive one LiDAR point cloud frame and generate the corresponding ground and non-ground point clouds. The standard deviation was calculated over the frames in each sequence after excluding warm-up frames. All runtime experiments were conducted on the same desktop PC listed in Table 1, and the same timing procedure was used for all compared methods.

Table 10 presents the processing time comparison. The proposed method achieved an average processing time of 12.15 ms per frame over sequences 00–10, which was lower than Patchwork++ (34.47 ms), JPC (31.32 ms), and GroundGrid (49.94 ms). The proposed method also showed a low average standard deviation of 5.12 ms, indicating stable processing time across frames.

The processing time of the proposed method ranged from 10.50 ms to 15.54 ms across the evaluated sequences. The fastest average processing time was observed on sequence 01, whereas the slowest average processing time was observed on sequence 09. Overall, the proposed method maintained a relatively consistent processing time across different sequences.

The short processing time of the proposed method is mainly due to its simple frame-wise processing structure. Unlike methods that maintain an elevation map or perform terrain interpolation, the proposed method uses only current-frame cell-wise height statistics, dual-seed initialization, neighbor-cell expansion, and point-level height-band refinement. Therefore, the proposed method can be used as a fast preprocessing module for LiDAR-based autonomous driving perception.

However, the processing time comparison should be interpreted with caution. Although the same spatial input range was used by pre-cropping the input point clouds, the compared methods differ in their internal data representations, temporal-state usage, implementation details, and threading conditions. In particular, GroundGrid was evaluated using a multi-threaded configuration, whereas the proposed method was evaluated using a single-threaded implementation. Therefore, the processing time results should be regarded as an empirical comparison under the experimental setting used in this study rather than a strictly normalized benchmark.

**Figure 12:**
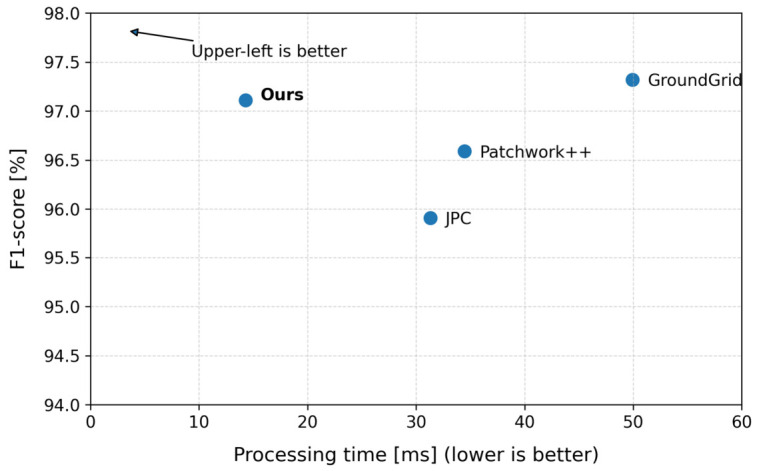
F1-score–runtime trade-off across different ground segmentation algorithms.

Figure 12 shows the accuracy–runtime trade-off across the compared ground segmentation algorithms. The proposed method achieved an F1-score of 97.11% with an average processing time of 12.15 ms per frame. Although GroundGrid achieved the highest F1-score of 97.32%, its average processing time was 49.94 ms per frame under the experimental setting used in this study. Compared with Patchwork++ and JPC, the proposed method achieved a higher F1-score while requiring a shorter processing time. These results indicate that the proposed method provides a favorable balance between segmentation accuracy and computational efficiency for real-time LiDAR preprocessing.

## 4. Discussion

The experimental results show that the proposed method achieved stable ground segmentation performance while maintaining a simple grid-based processing structure. Although the average accuracy, F1-score, and ground IoU were lower than those of GroundGrid, the proposed method showed comparable performance to other representative ground segmentation methods. This result indicates that the dual-seed initialization strategy can provide reliable initial ground candidates without using temporal elevation-map updates or complex terrain interpolation.

The main advantage of the proposed method is its computational efficiency. The method uses only current-frame cell-wise height statistics, dual-seed initialization, neighbor-cell expansion, and local height-band refinement. Therefore, it does not require iterative plane fitting, learning-based inference, or maintenance of a temporal terrain map. This simple structure contributed to the short processing time observed in the experiment. In this sense, the proposed method is suitable for LiDAR preprocessing modules in which fast execution and implementation simplicity are more important than the highest segmentation accuracy.

The lower accuracy compared with GroundGrid can be explained by the difference in terrain modeling strategy. GroundGrid estimates terrain using an elevation-map-based framework with ground confidence estimation, interpolation, and point cloud segmentation. In contrast, the proposed method processes each frame independently and expands ground cells based on local height continuity. As a result, the proposed method can be more sensitive to abrupt height changes, vegetation boundaries, curbs, sparse distant points, and mixed cells containing both ground and non-ground points.

The qualitative results also support this interpretation. Road surfaces and flat ground regions were generally segmented correctly, whereas errors occurred near object boundaries, curbs, vegetation, and discontinuous terrain structures. The point-level height-band refinement reduced some mixed-cell errors, but it cannot fully resolve cases where ground and low-height objects have similar height distributions within the same grid cell. In addition, because the Cartesian grid uses a fixed cell size, the method does not fully reflect the distance-dependent sparsity of rotating LiDAR measurements.

The LiDAR-height-based seed assumes that the sensor mounting height is known and that the expected ground height can be approximated in the vehicle coordinate system. This assumption may become less accurate when the vehicle is strongly tilted, driving on steep ramps, affected by suspension motion, or when calibration errors exist. In such cases, the expected ground height may deviate from the actual local ground level. The proposed method alleviates this limitation by combining the LiDAR-height seed with the global height-distribution-based seed, but adaptive estimation of the sensor-to-ground relationship remains an important direction for future work.

Most of the remaining errors occurred near curbs, vegetation boundaries, object boundaries, and abrupt terrain changes. These errors are mainly caused by the fixed Cartesian grid resolution and the use of local height continuity during cell expansion. In mixed cells, ground and low-height non-ground points may have similar height distributions, making complete separation difficult using only height statistics. Sparse distant regions can also reduce the reliability of cell-wise statistics, which may lead to missed ground cells or inaccurate expansion.

Another limitation of this study is that the evaluation was conducted only on the SemanticKITTI dataset. Although SemanticKITTI is widely used for LiDAR point cloud evaluation, its data were collected using a specific sensor configuration and driving environment. In addition, vegetation points were excluded from the quantitative metrics following the evaluation setting used in GroundGrid. This is because the vegetation class in SemanticKITTI is not always clearly separable into ground and non-ground surfaces, making it difficult to determine whether the majority of vegetation-labeled points should be regarded as part of the ground surface. However, vegetation boundaries can still affect the segmentation result in practice. Therefore, additional evaluation on other datasets and a more detailed error analysis with respect to distance, point density, curbs, object boundaries, slopes, and vegetation regions are required to further verify the generalization capability of the proposed method.

Future work will focus on improving robustness in sparse and complex regions. Distance-adaptive thresholds, point-density-based correction, and a hybrid Cartesian-polar grid representation could be incorporated to reduce errors in far regions and near object boundaries. In addition, evaluating the proposed method on additional datasets and real vehicle experiments would further verify its generalization capability.

## 5. Conclusions

This paper proposed a dual-seed initialization method for Cartesian grid-based LiDAR ground segmentation. The proposed method selects initial ground candidate cells by combining a global seed based on the overall height distribution and a sensor seed based on the LiDAR mounting height. The ground region is then estimated through eight-neighbor cell expansion. In addition, a local height-band refinement process was applied to alleviate the mixed-cell problem, where ground and non-ground points coexist within the same grid cell.

Experiments using the SemanticKITTI dataset showed that the proposed method achieved an average accuracy of 96.34%, an F1-score of 97.11%, and an IoU(G) of 94.40%. Although its quantitative accuracy was lower than that of GroundGrid, the proposed method achieved an average processing time of 12.15 ms per frame over SemanticKITTI sequences 00–10, which was the shortest among the compared methods. These results indicate that the proposed method is suitable for LiDAR preprocessing environments that require a simple structure and fast processing speed, rather than the highest ground segmentation accuracy.

The main contribution of this study is not in constructing a complex terrain estimation model, but in maintaining the simplicity of a Cartesian grid-based structure while improving the stability of initial ground candidate selection and ensuring real-time applicability through dual-seed initialization and mixed-cell correction. Future work will focus on improving ground segmentation accuracy and robustness by compensating for sparse distant points, applying distance-adaptive thresholds, incorporating point-density-based correction, and combining the proposed method with a polar grid structure.

## Figures and Tables

**Table 1:** Experimental environment.

Item	Setting
CPU	Intel Core i9-9940X (Intel Corporation, Santa Clara, CA, USA)
OS	Ubuntu 18.04
Framework	ROS 1 Melodic Morenia
Implementation language	C++11
Dataset	SemanticKITTI
Evaluation sequences	00–10
Processing mode	Single-frame processing

**Table 2:** Parameters used in the experiments.

Parameter	Value	Description
ROI range in x-axis	−50 to 50 m	Region of interest in the longitudinal direction
ROI range in y-axis	−50 to 50 m	Region of interest in the lateral direction
Grid resolution, r	0.50 m	Cell size of the Cartesian grid
Height variance threshold, τ_var	0.001 m^2^	Maximum height variance for seed cell selection
Global seed height threshold, τ_g	0.15 m	Height tolerance from the global reference height
LiDAR mounting height, h_L	1.73 m	Mounted height of the LiDAR sensor
LiDAR seed height threshold, τ_L	0.50 m	Height tolerance from the expected ground height
Local slope threshold, τ_s	0.20	Maximum slope for neighbor-cell expansion
Mixed-cell point count threshold	3 points	Minimum point count for mixed-cell checking
Mixed-cell height range threshold, τ_m	0.4 m	Height range threshold for identifying mixed cells
Local ground height-band threshold, τ_b	0.10 m	Point-level height band for ground classification

**Table 3:** Quantitative comparison of ground segmentation performance on SemanticKITTI sequences 00–10.

Seq	00	01	02	03	04	05	06	07	08	09	10	Mean
Precision												
Patchwork++	94.99	98.27	95.96	96.81	98.18	92.65	97.86	93.29	96.97	96.06	92.81	95.80
JPC	96.78	97.97	97.50	98.09	99.01	94.03	97.96	95.65	97.97	97.64	95.27	97.08
GroundGrid	96.05	98.01	97.36	97.96	99.08	95.19	97.82	95.31	97.50	97.25	95.38	96.99
Ours	96.15	96.93	96.94	97.76	98.69	94.16	98.19	95.09	97.66	96.97	94.14	96.61
Recall												
Patchwork++	98.67	96.52	97.20	98.17	97.21	98.13	97.39	98.42	97.41	96.45	95.93	97.41
JPC	97.20	95.46	93.72	94.86	96.91	95.64	96.23	96.53	95.13	92.66	88.47	94.97
GroundGrid	98.70	96.17	97.71	97.95	97.85	98.13	98.38	98.72	97.79	96.91	95.90	97.65
Ours	98.93	96.72	97.66	98.33	98.38	98.12	97.83	98.53	97.70	96.37	95.38	97.63
F1-score												
Patchwork++	96.80	97.39	96.58	97.49	97.69	95.31	97.63	95.79	97.19	96.25	94.35	96.59
JPC	96.99	96.70	95.57	96.45	97.95	94.83	97.09	96.09	96.53	95.09	91.74	95.91
GroundGrid	97.35	97.08	97.54	97.96	98.46	96.64	98.10	96.99	97.64	97.08	95.64	97.32
Ours	97.52	96.82	97.30	98.05	98.53	96.10	98.01	96.78	97.68	96.67	94.76	97.11
Accuracy												
Patchwork++	96.64	95.96	95.08	96.08	96.39	94.79	96.63	95.88	96.37	94.90	93.75	95.68
JPC	96.89	94.93	93.80	94.59	96.81	94.37	95.89	96.26	95.60	93.50	91.35	94.91
GroundGrid	97.24	95.50	96.48	96.84	97.60	96.32	97.29	97.08	96.97	96.05	95.25	96.60
Ours	97.41	95.05	96.13	96.97	97.70	95.70	97.17	96.88	97.01	95.49	94.26	96.34
IoU												
Patchwork++	93.79	94.90	93.38	95.09	95.49	91.04	95.36	91.91	94.53	92.78	89.30	93.41
JPC	94.15	93.61	91.52	93.14	95.98	90.16	94.34	92.47	93.29	90.64	84.75	92.19
GroundGrid	94.84	94.33	95.19	96.00	96.97	93.49	96.27	94.15	95.40	94.33	91.64	94.78
Ours	95.16	93.84	94.74	96.17	97.11	92.49	96.10	93.76	95.46	93.55	90.04	94.40

Note: The results for Patchwork++, JPC, and GroundGrid are cited from Steinke et al. [13]. The results for the proposed method were obtained in this study.

**Table 4:** Ablation study of the proposed method on SemanticKITTI sequences 00–10.

Variant	ACC	Precision	Recall	F1-Score	IoU(G)
Global seed only	90.31	96.92	87.63	91.88	85.20
LiDAR-height seed only	95.93	96.64	96.96	96.79	93.80
Dual seed without neighbor expansion	85.73	99.78	78.12	87.57	77.99
Dual seed without mixed-cell refinement	75.07	72.26	98.91	83.20	71.70
Proposed method	96.34	96.61	97.63	97.11	94.40

**Table 5:** Sensitivity to Grid Resolution.

r [m]	F1	IoU(G)	Processing Time [ms]
0.3	96.61	93.47	13.63
0.4	96.68	93.60	12.86
0.5	96.67	93.57	12.15
0.6	96.56	93.37	11.99
0.7	96.41	93.08	11.34

**Table 6:** Sensitivity to LiDAR-Height Seed Threshold.

τL [m]	F1	IoU(G)
0.4	97.09%	94.36%
0.45	97.11%	94.41%
**0.5**	97.12%	94.43%
0.55	97.12%	94.42%
0.6	97.10%	94.39%

**Table 7:** Sensitivity to Local Slope Threshold.

τs	F1	IoU(G)
0.10	96.91	94.04
0.15	96.96	94.12
0.20	97.04	94.26
0.25	97.03	94.25
0.30	96.98	94.16

**Table 8:** Sensitivity to Mixed-Cell Height Range Threshold.

τm [m]	F1	IoU(G)
0.30	97.12	94.42
0.35	97.17	94.51
0.40	97.19	94.55
0.45	97.18	94.53
0.50	97.15	94.48

**Table 9:** Sensitivity to Local Ground Height-Band Threshold.

τb [m]	F1	IoU(G)
0.06	97.00	94.20
0.08	97.24	94.65
0.10	97.25	94.67
0.12	97.19	94.55
0.14	97.08	94.35

**Table 10:** Processing time comparison on SemanticKITTI sequences 00–10.

Seq	00	01	02	03	04	05	06	07	08	09	10	Mean
Processing time [ms]												
Patchwork++	33.84	31.85	34.85	35.24	35.53	34.87	34.61	33.71	35.12	35.04	34.61	34.47
JPC	30.55	31.28	30.25	31.27	30.27	31.52	31.39	30.99	31.96	32.66	31.35	31.32
GroundGrid	45.75	41.37	51.48	48.30	50.93	45.93	52.39	53.90	54.77	49.50	55.00	49.94
Ours	11.08	10.50	12.81	11.95	12.71	15.54	13.27	11.59	11.46	11.44	11.32	12.15
Standard Deviation [ms]												
Patchwork++	7.15	7.86	7.16	7.06	6.50	7.33	7.75	7.22	6.93	7.04	7.67	7.24
JPC	6.97	8.19	7.24	7.09	7.25	7.51	6.60	7.17	6.79	7.06	7.01	7.17
GroundGrid	4.36	4.96	6.32	5.99	6.37	3.84	6.39	5.52	4.89	6.23	5.16	5.46
Ours	5.72	4.87	4.40	5.19	4.88	5.96	4.58	4.53	4.43	5.87	5.86	5.12

## Data Availability

No new data were created in this study. The data analyzed in this study are publicly available in the SemanticKITTI dataset cited in Reference [17].

## Abbreviations

The following abbreviations are used in this manuscript:

LiDAR	Light Detection and Ranging
ROI	Region of Interest
ROS	Robot Operating System
IoU	Intersection over Union
TIN	Triangulated Irregular Network
CPU	Central Processing Unit
TP	True Positive
FP	False Positive
TN	True Negative
FN	False Negative
JPC	Jump Point Convolution

## References

[1] Li Y., Ibanez-Guzman J. (2020). LiDAR for Autonomous Driving: The Principles, Challenges, and Trends for Automotive LiDAR and Perception Systems. IEEE Signal Process. Mag..

[2] Gomes T., Matias D., Campos A., Cunha L., Roriz R. (2023). A Survey on Ground Segmentation Methods for Automotive LiDAR Sensors. Sensors.

[3] Himmelsbach M., Hundelshausen F.v., Wuensche H.-J. Fast Segmentation of 3D Point Clouds for Ground Vehicles. Proceedings of the IEEE Intelligent Vehicles Symposium.

[4] Douillard B., Underwood J., Kuntz N., Vlaskine V., Quadros A., Morton P., Frenkel A. On the Segmentation of 3D LiDAR Point Clouds. Proceedings of the IEEE International Conference on Robotics and Automation.

[5] Zermas D., Izzat I., Papanikolopoulos N. Fast Segmentation of 3D Point Clouds: A Paradigm on LiDAR Data for Autonomous Vehicle Applications. Proceedings of the IEEE International Conference on Robotics and Automation.

[6] Chen T., Dai B., Wang R., Liu D. (2014). Gaussian-Process-Based Real-Time Ground Segmentation for Autonomous Land Vehicles. J. Intell. Robot. Syst..

[7] Moosmann F., Pink O., Stiller C. Segmentation of 3D LiDAR Data in Non-Flat Urban Environments Using a Local Convexity Criterion. Proceedings of the IEEE Intelligent Vehicles Symposium.

[8] Axelsson P. (2000). DEM Generation from Laser Scanner Data Using Adaptive TIN Models. Int. Arch. Photogramm. Remote Sens..

[9] Bogoslavskyi I., Stachniss C. Fast Range Image-Based Segmentation of Sparse 3D Laser Scans for Online Operation. Proceedings of the IEEE/RSJ International Conference on Intelligent Robots and Systems.

[10] Wu B., Wan A., Yue X., Keutzer K. SqueezeSeg: Convolutional Neural Nets with Recurrent CRF for Real-Time Road-Object Segmentation from 3D LiDAR Point Cloud. Proceedings of the IEEE International Conference on Robotics and Automation.

[11] Milioto A., Vizzo I., Behley J., Stachniss C. RangeNet++: Fast and Accurate LiDAR Semantic Segmentation. Proceedings of the IEEE/RSJ International Conference on Intelligent Robots and Systems.

[12] Cortinhal T., Tzelepis G., Aksoy E.E. SalsaNext: Fast, Uncertainty-Aware Semantic Segmentation of LiDAR Point Clouds. Proceedings of the International Symposium on Visual Computing.

[13] Steinke N., Goehring D., Rojas R. (2024). GroundGrid: LiDAR Point Cloud Ground Segmentation and Terrain Estimation. IEEE Robot. Autom. Lett..

[14] Cheng J., He D., Lee C. A Simple Ground Segmentation Method for LiDAR 3D Point Clouds. Proceedings of the International Conference on Advances in Computer Technology, Information Science and Communications.

[15] Fankhauser P., Hutter M. (2016). A Universal Grid Map Library: Implementation and Use Case for Rough Terrain Navigation. Robot Operating System.

[16] Geiger A., Lenz P., Stiller C., Urtasun R. (2013). Vision Meets Robotics: The KITTI Dataset. Int. J. Robot. Res..

[17] Behley J., Garbade M., Milioto A., Quenzel J., Behnke S., Stachniss C., Gall J. SemanticKITTI: A Dataset for Semantic Scene Understanding of LiDAR Sequences. Proceedings of the IEEE/CVF International Conference on Computer Vision.

[18] Shen Z., Liang H., Lin L., Wang Z., Huang W., Yu J. (2021). Fast Ground Segmentation for 3D LiDAR Point Cloud Based on Jump-Convolution-Process. Remote Sens..

[19] Lim H., Oh M., Myung H. (2021). Patchwork: Concentric Zone-Based Region-Wise Ground Segmentation with Ground Likelihood Estimation Using a 3D LiDAR Sensor. IEEE Robot. Autom. Lett..

[20] Lee S., Lim H., Myung H. Patchwork++: Fast and Robust Ground Segmentation Solving Partial Under-Segmentation Using 3D Point Cloud. Proceedings of the IEEE/RSJ International Conference on Intelligent Robots and Systems.

[21] Zhang Y., Zhou Z., David P., Yue X., Xi Z., Gong B., Foroosh H. PolarNet: An Improved Grid Representation for Online LiDAR Point Clouds Semantic Segmentation. Proceedings of the IEEE/CVF Conference on Computer Vision and Pattern Recognition.

[22] Paigwar A., Erkent Ö., Sierra-Gonzalez D., Laugier C. GndNet: Fast Ground Plane Estimation and Point Cloud Segmentation for Autonomous Vehicles. Proceedings of the IEEE/RSJ International Conference on Intelligent Robots and Systems.

[23] He D., Abid F., Kim Y., Kim J. (2022). SectorGSnet: Sector Learning for Efficient Ground Segmentation of Outdoor LiDAR Point Clouds. IEEE Access.

[24] Qi C.R., Su H., Mo K., Guibas L.J. PointNet: Deep Learning on Point Sets for 3D Classification and Segmentation. Proceedings of the IEEE Conference on Computer Vision and Pattern Recognition.

[25] Qi C.R., Yi L., Su H., Guibas L.J. (2017). PointNet++: Deep Hierarchical Feature Learning on Point Sets in a Metric Space. Adv. Neural Inf. Process. Syst..

[26] Thomas H., Qi C.R., Deschaud J.-E., Marcotegui B., Goulette F., Guibas L.J. KPConv: Flexible and Deformable Convolution for Point Clouds. Proceedings of the IEEE/CVF International Conference on Computer Vision.

[27] Hu Q., Yang B., Xie L., Rosa S., Guo Y., Wang Z., Trigoni N., Markham A. RandLA-Net: Efficient Semantic Segmentation of Large-Scale Point Clouds. Proceedings of the IEEE/CVF Conference on Computer Vision and Pattern Recognition.

[28] Haidar S.A., Al Khatib E.I., Popović M., Azzam R., Laugier C. (2025). HARP-NeXt: High-Speed and Accurate Range-Point Fusion Network for 3D LiDAR Semantic Segmentation. arXiv.

